# Seroprevalence of *Toxoplasma gondii* in goats in Hunan province, China

**DOI:** 10.1051/parasite/2016053

**Published:** 2016-10-20

**Authors:** Fen Li, Shi-Ping Wang, Chang-Jian Wang, Shi-Cheng He, Xiang Wu, Guo-Hua Liu

**Affiliations:** 1 Xiangya School of Medicine, Central South University Changsha Hunan province 410013 PR China; 2 College of Veterinary Medicine, Hunan Agricultural University Changsha Hunan province 410128 PR China; 3 Hunan Co-Innovation Center of Animal Production Safety Changsha Hunan province 410128 PR China; 4 Animal Disease Prevention and Control Center of Hunan province 410001 PR China

**Keywords:** *Toxoplasma gondii*, Seroprevalence, Goats, Indirect hemagglutination assay, Subtropical China

## Abstract

*Toxoplasma gondii* infections are prevalent in animals and humans worldwide. In the present investigation, the seroprevalence of *T. gondii* in goats was investigated in Hunan province, subtropical China between March 2014 and December 2015. A total of 1,028 serum samples collected from 14 administrative regions of Hunan province were evaluated by the indirect hemagglutination test (IHAT) for the detection of specific antibodies. Antibodies to *T. gondii* were detected in 124 serum samples (12%). The *T. gondii* seroprevalence ranged from 1.7% to 19% among different regions in subtropical China, and the differences were statistically significant (*p* < 0.01). The results of the present survey indicated that *T. gondii* infection is prevalent in goats in Hunan, which poses a potential risk for human infection with *T. gondii* in this province.

## Abstract (fr)

Les infections par *Toxoplasma gondii* sont répandues chez les animaux et les humains dans le monde entier. Dans la présente enquête, la séroprévalence de *T. gondii* chez les chèvres a été étudiée dans la province du Hunan, en Chine subtropicale, entre mars 2014 et décembre 2015. Un total de 1028 échantillons de sérum prélevés dans 14 régions administratives de la province du Hunan ont été évaluées par le test d’hémagglutination indirecte (IHAT) pour la détection d’anticorps spécifiques. Les anticorps dirigés contre *T. gondii* ont été détectés dans 124 échantillons de sérum (12 %). La séroprévalence de *T. gondii* a varié de 1.7 % à 19 % entre les différentes régions en Chine subtropicale, et les différences étaient statistiquement significatives (*p* < 0.01). Les résultats de la présente étude indiquent que l’infection par *T. gondii* est très répandue chez les chèvres dans le Hunan, ce qui pose un risque potentiel pour l’infection humaine par *T. gondii* dans cette province.

## Introduction

Toxoplasmosis is a zoonotic parasitic disease caused by *Toxoplasma gondii*, which infects humans and has a worldwide distribution [20]. It has been estimated that approximately one third of the world’s population has been infected with *T. gondii* [8]. It may cause abortion in pregnant women or occasionally toxoplasmic encephalitis or even death in patients with immune-suppression diseases like AIDS, although almost all infected people are asymptomatic [5]. In addition, *T. gondii* can also infect almost all warm-blooded animals [15].

In goats, the main clinical signs of toxoplasmosis are abortion and perinatal death, causing huge economic losses to the goat industry worldwide [1, 4]. In spite of the high seroprevalence of *T. gondii* reported in goats around the world [3, 6, 10], little information is available on the seroprevalence of *T. gondii* in goats in China [9, 18, 19, 21]. In the People’s Republic of China, the goat industry constitutes a large agricultural sector and is important in economic development. In addition, in China, goat meat is the most widely produced and consumed meat. Hunan province is one of the largest producers of goats in China. Although *T. gondii* infection causes major economic losses in goats, its prevalence in subtropical China might be underestimated and neglected. It has yet to be determined whether *T. gondii* infection is present in goats in Hunan province, subtropical China. Therefore, investigation of *T. gondii* infection in goats has important implications for the prevention and control of *T. gondii* infection in animals and humans in this province of subtropical China.

Given this background and the zoonotic significance of *T. gondii*, the objective of the present investigation was to examine the seroprevalence (animals-level) of *T. gondii* infection in goats in Hunan province. The results should provide a foundation for the implementation of control strategies against *T. gondii* infection in goats in this province and elsewhere.

## Materials and methods

Hunan province is situated in the central eastern part of mainland China, between the northern latitudes of 24°38′–30°08′ and eastern longitudes of 108°47′–114°15′. The surface area is 211,800 square km, with a population of more than 71 million. Hunan’s climate is subtropical: January temperatures average 3–8 °C while July temperatures average around 27–30 °C. The average annual rainfall ranges from 1,200 to 1,700 mm. Hunan province is divided into 14 administrative regions (cities), with the city of Changsha as its capital.

A total of 1,028 blood samples were collected from 54 intensive farms in Hunan province between March 2014 and December 2015. The numbers of goats reared on each farm ranged from approximately 100 to 1,000. The serum samples cover almost the whole Hunan province. Healthy goats were randomly selected for blood samples. Samples were then centrifuged at 1,000 g for 10 min, and the serum was collected, frozen, and stored at −20 °C until it was assayed.

Serum samples were tested for antibodies against *T. gondii* by the indirect hemagglutination test (IHAT) viable kit (NY/T 573-2002, Lanzhou Veterinary Research Institute, Chinese Academy of Agricultural Sciences) according to the manufacturer’s instructions. The kit is commercially available and has been used for many years in China to detect specific antibodies to *T. gondii* in goats and other mammals [7, 11, 13, 17]. The serum samples were identified as positive if an agglutination reaction was seen in wells with dilutions of 1:64 or higher.

A multivariable mixed-effects logistic regression model with the farm as a random effect was used. Other variables were introduced as fixed effects in the model. The data were analyzed statistically using the PASW Statistics 18 program (IBM Corporation, Somers, NY); 95% confidence intervals (CI) were given. The value of *p* < 0.05 was considered statistically significant in the multivariate analysis.

All animals were handled in strict accordance with good animal practices according to the Animal Ethics Procedures and Guidelines of the People’s Republic of China, and the study was approved by the Animal Ethics Committee of Central South University.

## Results and discussion

Limited information is available for *T. gondii* infections in goats in China [9, 18, 19, 21]. No survey of the seroprevalence of *T. gondii* in goats in tropical China has been reported. IHAT is a simple technique for detecting *T. gondii* antibodies (IgG and IgM) and has been used extensively in many animals in China [7, 11, 13, 17]. Therefore, the present study used IHAT to detect *T. gondii* antibodies in goats using a commercially marketed kit.

In the present study, antibodies against *T. gondii* were detected in 12.1% of goats (124/1028; 95% CI: 10–14.1). The *T. gondii* seroprevalence in goats from different regions ranged from 1.7% (95% CI: 0–5.1) to 18.8% (95% CI: 10.2–27.3) (Table 1), having statistically significant differences (*p* < 0.01). This seroprevalence was similar to that reported in goats in Yunnan province (11.9%) [21] and in northwestern China (14.1%) [9], but was lower than that in northeastern China (16.9%) [19] and Qinghai province (29.5%) [18]. Differences in *T. gondii* seroprevalence are likely due to differences in animal welfare, climates, and husbandry practices. Results of the present and previous investigations [9, 18, 19, 21] indicate that *T. gondii* infection is widespread in goats in China.

**Table 1.** Seroprevalence of *Toxoplasma gondii* in goats in Hunan province, subtropical China.

Region	No. tested	No. positive	Prevalence (%)	95% CI
Changsha	52	3	5.8	0–12.1
Zhuzhou	50	3	6	0–12.6
Xiangtang	100	12	12	5.6–18.4
Hengyang	60	7	11.7	3.5–19.8
Shaoyang	90	12	13.3	6.3–20.4
Yueyang	58	1	1.7	0–5.1
Chande	70	13	18.6	9.5–27.7
Zhangjiajie	80	15	18. 8	10.2–27.3
Yiyang	60	3	5	0–10.5
Loudi	41	1	2.4	0–7.2
Chenzhou	85	10	11.8	4.9–18.6
Yongzhou	50	4	8	0.5–15.5
Huaihua	50	9	18	7.4–28.6
Xiangxi	182	31	17	11.6–22.5
Total	1028	124	12.1	10.1–14.1

Female goats (12.4%; 95% CI: 10.3–14.6) had higher *T. gondii* seroprevalence than males (9.9%; 95% CI: 5.1–14.6) (*p* > 0.05). This finding indicates that *T. gondii* infection is more likely in females than males. *T. gondii* seroprevalence in goats was higher in autumn (15.4%; 95% CI: 11.7–19.1), followed by winter (11.4%; 95% CI: 5.3–17.5), but lower in summer (11.3%; 95% CI: 8.4–14) and spring (1.4%; 95% CI: 0–4.2) (Table 2). These results suggest that *T. gondii* infection in goats is prevalent all year round. This is likely to be associated with the moist and warm climate in tropical China, which is favorable for survival of the oocysts [14]. The highest prevalence of *T. gondii* was found in goats older than 4 years (18.2%; 95% CI: 9.6–26.8), followed by goats < 1 year old (15.8%; 95% CI: 11.1–20.5). *T. gondii* prevalence in different ages of goats ranged from 8.3% (95% CI: 5.2–11.3) to 18.2% (95% CI: 9.6–26.8) (Table 2) (*p* > 0.05). The present study indicated that age is a predisposing factor for *T. gondii* infection, consistent with results in other animals [12, 16]. The seroprevalence of *T. gondii* increases with growth in goats, indicating that there may be a cumulative likelihood for exposure to *T. gondii* infection with age.

**Table 2.** Seroprevalence of *Toxoplasma gondii* in goats by gender, season, and age in Hunan province, subtropical China.

Factor	Category	No. tested	No. positive	Prevalence (%)	95% CI
Gender	Male	152	15	9.9	5.1–14.6
	Female	876	109	12.4	10.3–14.6
Season	Spring	70	1	1.4	0–4.2
	Summer	489	55	11.3	8.4–14
	Autumn	364	56	15.4	11.8–19.1
	Winter	105	12	11.4	5.3–17.5
Age	<1 year	228	36	15.8	11.1–20.5
	1 ≤ years <2	420	49	11.7	8.6–14.7
	2 ≤ years <3	303	25	8.3	5.2–11.3
	≥4 years	77	14	18.2	9.6–26.8
	Total	1028	124	12.1	10.1–14.1

Humans can become infected by *T. gondii* through ingestion of oocyst-contaminated food, water, or undercooked meat. The present results reveal the presence of *T. gondii* infection in goats in tropical China, indicating contamination of the environment by *T. gondii* oocysts, which poses a risk of human infection with *T. gondii*. As a result, further work is required to assess whether the soil and water on goat farms or in other regions in tropical China are also contaminated by *T. gondii* oocysts [2]. In addition, toxoplasmosis can lead to abortion, stillbirth, and mummification in pregnant goats [1, 4]. However, the present dataset could not determine whether or not *T. gondii* infection can significantly increase the risk of abortion in goats in tropical China. Therefore, further studies are necessary to investigate a potential effect of *T. gondii* on reproduction in goats.

## Conclusions

The results of the present survey indicate that *T. gondii* infection is prevalent in goats in subtropical China. Therefore, it is imperative to implement integrated control strategies and measures to prevent and control *T. gondii* infection in goats. This is the first time that infection with *T. gondii* in goats has been reported in Hunan province.
